# Mitigating the unintended consequences of health-care initiatives

**DOI:** 10.2471/BLT.25.020125

**Published:** 2025-01-01

## Abstract

Health-care policies and initiatives are designed to save lives and enhance well-being, but they can also entrain unintended negative effects. Gary Humphreys reports.

“It often starts with a great idea,” says Kathryn Oliver. “People, by which I mean academics like me, tend to get carried away with a good, implementable idea without thinking through how it is supposed to bring about change or what impact it might have.”

Professor of Evidence and Policy at the London School of Hygiene and Tropical Medicine in the United Kingdom of Great Britain and Northern Ireland, Oliver has spent much of the past 10 years looking at the unintended adverse consequences (UAC) of health initiatives and exploring ways to limit their occurrence.

Broadly defined as outcomes that derive from initiatives that are not foreseen or intended, UAC occur across the health-service spectrum, from system-wide health financing reforms to targeted behaviour-change interventions.

To get a clearer understanding of why they occur, in collaboration with colleagues, Oliver organized a workshop with policy-makers and researchers in the United Kingdom in 2019. The findings of that consultation became the basis of CONSEQUENT, a framework designed to address the issue of UAC that was released in 2024.

“The workshop highlighted three main reasons for the occurrence of UAC,” Oliver explains. “Poor initiative design; a lack of clarity about goals or how they are to be achieved; and the misuse or misinterpretation of evidence.”

According to Oliver, poor design can take many forms but often derives from a desire to focus on one – usually quantifiable – aspect of what are generally composite, complex health issues. Among the examples she cites are anti-obesity initiatives focused on calorie counting.

“There have been a number of such initiatives in the United Kingdom and elsewhere in which students are encouraged to count the calories they are consuming and to mark them down in a diary,” she says. “Other initiatives have involved weighing students and sending body mass index report cards to parents. Both have been linked to increased bullying, body-image issues, and the development of eating disorders among children and adolescents.”

“We […] need to embrace complexity.”

Kathryn Oliver

A similar tendency is observable in initiatives designed to boost health system performance, as borne out by a study published in the May 2022 issue of *BMC Health Services Research.* The study describes the ways in which the use of contracts, targets and scorecards to improve performance inadvertently created “measure fixation”, focusing on improving the measurable aspects of performance rather than the underlying quality or purpose of the work.

Oliver points to silo-thinking driven by academic specialization or programme-driven agendas as a possible driver of narrowly focused initiatives. “In my experience, government policy-makers are more likely to think in terms of trade-offs, whereas academics and programme managers can be quite narrowly focused, especially when their funding is linked to achieving certain targets,” she notes.

For Prashant Yadav, a senior fellow for global health at the Council on Foreign Relations and an expert in health-care supply chains, one-size-fits-all solutions are also a concern. “They fail to account for the nuances of local contexts, infrastructure and demand patterns,” he says, citing the example of min-max restocking initiatives implemented in sub-Saharan Africa.

The approach involves maintaining stock levels between a predetermined minimum, to avoid stockouts, and a maximum, to prevent overstocking and wastage. Monthly replenishment quantities are calculated as the difference between the target maximum stock level and the sum of current on-hand and pipeline inventory.

According to Yadav, the approach has been promoted and supported by nongovernmental organizations who train staff in health-care clinics to use it. Problems have arisen when it is used for antimalarials, because it ignores seasonal variations, local disease patterns and logistical challenges. “Training staff to use min-max has given rise to chronic mismatches in supply, severe shortages in some facilities and overstocking in others,” he says.

Failing to take account of local conditions is flagged by Oliver as one of the key drivers of UACs.

It is also singled out by Professor Adnan Qadir Khan, Professor in Practice at the London School of Economics and Political Science. Khan specializes in public policy, state capacity and economic development, and has made a particular study of initiatives developed in high-income countries and then implemented in lower-income countries. The social distancing measures introduced by most countries during the coronavirus disease 2019 (COVID-19) pandemic are one example.

“They fail to account for the nuances of local contexts.”

Prashant Yadav

“The impact of social distancing measures, especially stay-at-home orders, differed significantly depending on context, notably in terms of economic activity. In rich countries, the trade-off between lives and livelihoods is not a core consideration because governments can subsidize people’s incomes, and many people can work from home,” he says. “This is less true for low-income countries, where trade-offs are much starker due to economic vulnerability, food insecurity, informality and limited fiscal space for subsidization.”

Khan emphasizes the importance of grounding policies and the initiatives in the local context, and highlights the importance of strengthening local institutions and human capital to effectively design and implement policies.

While adapting initiatives to reflect local conditions may seem like an obvious proposition, it is by no means straightforward, and has been the subject of controversy in the past. The differentiated approaches to breastfeeding recommended by the World Health Organization (WHO) at the beginning of the human immunodeficiency virus (HIV) pandemic is a case in point.

Prior to the introduction of antiretroviral therapies, in resource-limited settings where there was a risk of exposure to waterborne diseases, WHO recommended continuing breastfeeding. In settings where clean water and safe alternatives to breastfeeding were accessible (i.e. contaminant-free formula), replacement feeding was recommended to avoid HIV transmission.

As noted by Dr Glenda Gray (see interview), the recommendations were perceived as unfair. In the wake of a study focused on empowering women to make informed choices about how to feed their babies, Gray and her colleagues worked with women in Soweto, South Africa, explaining the risks and supporting those who chose formula feeding by providing clean water, proper techniques and formula supplies.

The way initiatives are perceived has only increased in relevance and influence, with the advent of social media, misinformation and the politicization of health care, feeding into a public health space that has never been more complex.

For Oliver, it is the complexity of the public health space in which public health policy is supposed to work that makes the drawing up and implementation of policy so uniquely challenging. “There are multiple dynamics in play, and it is not always easy to see how a given input will bring about change, while the outputs are often surprising,” she says.

A prime example of this is people fishing with insecticide-treated mosquito nets, a practice that has been documented in several studies, including studies done in the United Republic of Tanzania and Mozambique. Not only does the repurposing take a net away from someone who needs protection, the nets’ fine mesh picks up juvenile fish, crucial to the sustainability of fish stocks. There are also concerns about insecticides leaching into bodies of water, leading to unintended environmental consequences.

It is partly to capture these sorts of “surprises” that pilot programmes have become a critical step in developing, refining and scaling public health interventions, allowing researchers and policy-makers to evaluate feasibility, effectiveness and cultural acceptability in targeted communities before broader implementation. Ideally, once scaled, initiatives are monitored, and adjustments made where necessary.

While acknowledging the value of pilots and iterative adjustment based on monitoring, Oliver believes there is scope for more methodical approaches to mitigating unintended consequences, notably in the early phases of initiative formation, and taking full account of the way a given initiative is to bring about change.

It was to support such approaches that she and a group of colleagues developed the CONSEQUENT framework. The framework incorporates elements from the WHO-INTEGRATE framework, an evidence-to-decision framework developed by WHO to facilitate structured reflection and discussion from the beginning to the completion of a guideline development or other health decision-making process. It also draws on elements from the Behaviour Change Wheel, a framework developed in 2011 that identifies capability**, **opportunity and motivation as drivers of behaviour.

“CONSEQUENT guides users through several steps, beginning with the creation of a logic model to map how the intervention operates within its specific context. It then prompts the identification of potential unintended consequences and the mechanisms likely to trigger them,” Oliver explains. “Users are encouraged to map affected populations, review existing literature to uncover causal pathways and risks, and engage stakeholders including critics to gain diverse insights.” The process is iterative, allowing for continuous refinement as new information becomes available.

CONSEQUENT was launched in 2024. It remains to be seen whether it will be taken up. Oliver hopes that it will. “If we want initiatives that truly make a difference, we need to be clear about how what we’re doing will bring about change,” she says. “We also need to embrace complexity, listen to those affected, and question everything – even our best intentions.”

**Figure:**
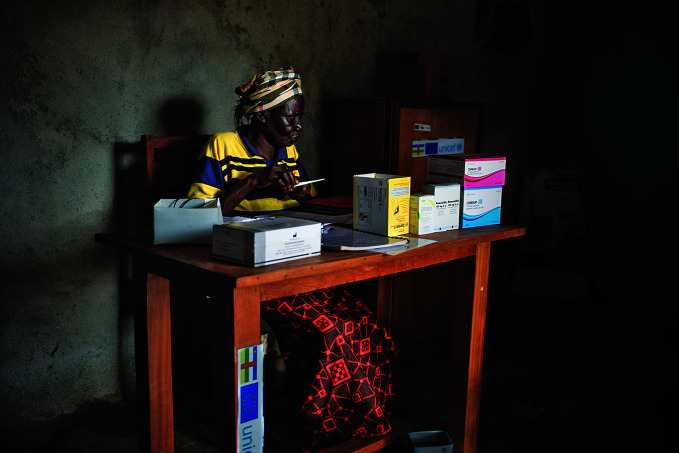
A community health worker prepares antimalarial medications for distribution in the Central African Republic

UNICEF/ Florent Vergnes

**Figure:**
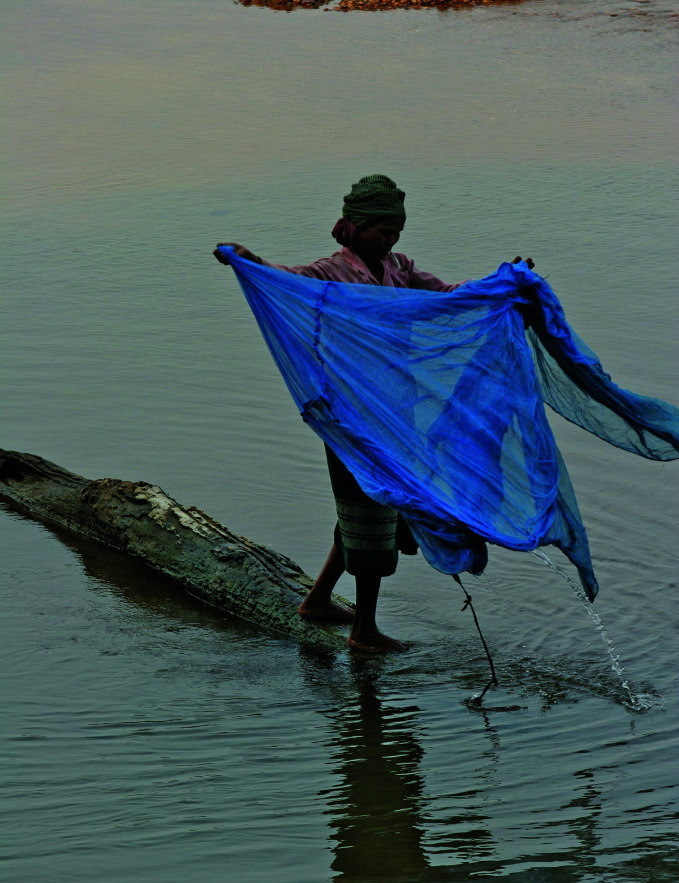
A woman washes an insecticide-treated bednet in the Se sang River in Phak Nam, Cambodia

WHO/Stephenie Hollyman

